# Antiviral Effect of Ginsenosides rk1 against Influenza a Virus Infection by Targeting the Hemagglutinin 1-Mediated Virus Attachment

**DOI:** 10.3390/ijms24054967

**Published:** 2023-03-04

**Authors:** Xia Yang, Hailiang Sun, Zhening Zhang, Weixin Ou, Fengxiang Xu, Ling Luo, Yahong Liu, Weisan Chen, Jianxin Chen

**Affiliations:** 1 Guangdong Provincial Key Laboratory of Veterinary Pharmaceutics Development and Safety Evaluation, South China Agricultural University, Guangzhou 510642, China; 2 College of Veterinary Medicine, South China Agricultural University, Guangzhou 510642, China; 3 Department of Biochemistry and Genetics, La Trobe Institute for Molecular Science, La Trobe University, Melbourne, VIC 3086, Australia

**Keywords:** ginsenosides, ginsenoside rk1, influenza virus, entry inhibitors, hemagglutinin

## Abstract

Influenza A virus (IAV) infections have been a serious hazard to public health everywhere. With the growing concern of drug-resistant IAV strains, there is an urgent need for novel anti-IAV medications, especially those with alternative mechanisms of action. Hemagglutinin (HA), an IAV glycoprotein, plays critical roles in the early stage of virus infection, including receptor binding and membrane fusion, making it a good target for developing anti-IAV drugs. Panax ginseng is a widely used herb in traditional medicine with extensive biological effects in various disease models, and its extract was reported to show protection in IAV-infected mice. However, the main effective anti-IAV constituents in panax ginseng remain unclear. Here, we report that ginsenoside rk1 (G-rk1) and G-rg5, out of the 23 screened ginsenosides, exhibit significant antiviral effects against 3 different IAV subtypes (H1N1, H5N1, and H3N2) in vitro. Mechanistically, G-rk1 blocked IAV binding to sialic acid in a hemagglutination inhibition (HAI) assay and an indirect ELISA assay; more importantly, we showed that G-rk1 interacted with HA1 in a dose-dependent manner in a surface plasmon resonance (SPR) analysis. Furthermore, G-rk1 treatment by intranasal inoculation effectively reduced the weight loss and mortality of mice challenged with a lethal dose of influenza virus A/Puerto Rico/8/34 (PR8). In conclusion, our findings reveal for the first time that G-rk1 possesses potent anti-IAV effects in vitro and in vivo. We have also identified and characterized with a direct binding assay a novel ginseng-derived IAV HA1 inhibitor for the first time, which could present potential approaches to prevent and treat IAV infections.

## 1. Introduction

Influenza A virus (IAV), a single-stranded RNA virus belonging to the Orthomyxoviridae family, is the cause of influenza, a contagious and acute respiratory disease accompanied by fever [1]. IAV has a broad host range and comprises many subtypes [2], causing severe health problems, mortality, and socio-economic losses globally. Seasonal IAV infections result in 290,000 to 650,000 fatalities yearly worldwide [3]. Outbreaks of highly pathogenic avian influenza (HPAI), such as H5N1 (1997 and 2003), H7N7 (2003), and H7N9 (2013), have resulted in alarmingly high fatality rates [4,5,6]. Vaccines and drugs are the two main approaches to prevent and treat IAV infections. However, IAV vaccines have many limitations that affect their efficacy, including the potential genetic mismatching between virus strains used in the vaccines and those circulating and in the elderlies with reduced or compromised immunity. There are now three kinds of flu medications that have FDA approval: neuraminidase (NA) inhibitors (NAIs) (i.e., oseltamivir, zanamivir, and peramivir), matrix protein 2 (M2) inhibitors (amantadine and rimantadine) [7], and a cap-dependent endonuclease inhibitor (Baloxavir marboxil) [8]. Unfortunately, M2 ion channel inhibitors have very limited effect because of extensive resistance from clinically circulating strains [9]. Meanwhile, resistant IAV strains against NAIs and Baloxavir are also increasing [10,11,12]. Therefore, developing novel anti-IAV agents is of great importance. So far, the core targets included RNA-dependent RNA polymerase (RdRp), hemagglutinin (HA), neuraminidase (NA), and M2 proton channel [13]. However, since HA plays a critical role during viral entry, inhibition of HA means blocking the initial step of viral infection, which could be especially significant if conserved sites in HA are blocked to potentially have broad-spectrum anti-influenza effects [14]. Therefore, HA is an attractive target for the development of anti-influenza drugs.

Two phylogenetic groups—group 1 (H1, H2, H5, H6, H8, H9, H11, H12, H13, H16, H17, and H18) and group 2 (H3, H4, H7, H10, H14, and H15)—can be formed from the 18 distinct HA subtypes that have been reported [15]. In the form of a homotrimer, mature HA is essential for viral attachment and membrane fusion. The primary translation product, HA0, which has numerous glycosylations, is cleaved into two disulfide-linked polypeptides, HA1 (which contains the receptor binding site (RBS)) and HA2 (which contains the fusion domain), to form the HA monomer [16]. Recent preclinical studies have reported significant therapeutic effects of anti-HA neutralizing antibodies, such as C05, CR9114, CR6261, and CR8020, but there are still challenges with their manufacturing and supply [17,18,19]. Small compounds, as opposed to antibodies, offer great shelf stability and are relatively inexpensive to produce [20]. Therefore, developing novel HA inhibitors is urgently needed for controlling seasonal influenza and potential outbreaks of epidemics induced by newly emerging IAVs in the future.

Panax ginseng, a well-known herbal nutritional supplement commonly used in traditional medicine, is widely utilized in East Asian countries, such as China and Korea [21]. Currently, more and more attention is being paid to Panax species due to their extensive biological effects in various disease models. It contains a plethora of pharmacologically active compounds, including ginsenosides, polysaccharides, polyacetylenes, phytosterols, and essential oils. Over 280 ginsenosides have been identified from Panax species and are believed to be the primary bioactive components [22]. Ginsenosides have a wide range of biological actions, including anti-cancer, anti-cardiovascular disease, anti-obesity, anti-diabetes, and anti-central nervous system disorder effects, as well as enhancing strength and sexuality [23]. Additionally, ginseng extracts or ginsenosides were reported to have antiviral activities against human pathogenic virus infections, such as hepatitis (HAV and HBV), HIV-1, human herpes (HSV-1, HSV-2), and respiratory syncytial virus (RSV) [24,25,26,27,28]. Wang et al. reported that fermented ginseng extracts improved the survival of mice infected with various IAV strains, including H1N1, H3N2, and H7N9 [29]. Additionally, feeding mice and ferrets a diet rich in red ginseng prevented them from contracting the highly pathogenic H1N1 influenza virus, which can be fatal [30]. Dong et al. reported that ginsenoside rb1 (G-rb1) protected mice from lethal 2009 pandemic H1N1 infection. Moreover, G-rb1 inhibited IAV infection by preventing viral particles’ attachment to α 2-3′ sialic acid (SA) receptors on Chinese hamster ovary cells [31]. Unfortunately, G-rb1 administration after viral infection did not show any protection on 2009 panH1N1-infected mice, and this study also used a very high concentration of G-rb1 (450 µM) in vitro, indicating a likely weak interaction between G-rb1 and viral HA. However, ginseng extracts have shown significant antiviral effects against IAV infection in vivo [29,30]. Therefore, we speculated that there might be more effective antiviral constituents in ginseng, which would certainly deserve further exploration.

This study focused on identifying anti-IAV ginsenosides and the underlying antiviral mechanism. Our results demonstrate that G-rk1 and G-rg5 possess remarkable anti-IAV effects in IAV-infected A549 cells, and G-rk1 intranasal administration exhibited significant protection in IAV (PR8, H1N1)-infected mice. To the best of our knowledge, this is the first concrete proof that ginseng-derived G-rk1 binds HA1 and may act as an inhibitor of IAV entry.

## 2. Results

### 2.1. Cell Cytotoxicity and Inhibitory Effects of G-rk1 and G-rg5 against IAV Infection in A549 Cells

According to the quantity of hydroxyl and glycosidic linkages connecting the aglycone moiety to the ginsenoside, ginsenosides are divided into two groups: protopanaxadiol (PPD) and protopan-axatriol (PPT). The smaller and less polar ginsenosides were created during the steaming of ginseng through hydrolysis, dehydration, and isomerization at C-3, C-6, or C-20 [32]. To identify the antiviral properties of ginsenosides, the inhibitory effects of 14 PPDs (Figure S1) and 9 PPTs (Figure S2) at 10 μM against PR8 replication in A549 cells were evaluated using IFA. Ribavirin, a well-known inhibitor of viral RNA synthesis [33], is used as a positive control that significantly inhibits the replication of IAV PR8 (Figure S3). Out of the 23 compounds, 6 ginsenosides, including ginsenoside rg1, Mb, 20(S)-rg2, 20(R)-rg2, rg5, and rk1, exhibited significant inhibition of PR8 replication, reflected by reduced viral NP expression. Noticeably, less polar ginsenosides (20(S)-rg2, 20(R)-rg2, G-rk1, and G-rg5) exhibited stronger antiviral activity than ginsenosides rg1 and Mb, and G-rk1 and G-rg5 displayed the strongest inhibition (Figure S4). The CC_50_ and EC_50_ values were determined, as shown in Table S1.

**Figure 1:**
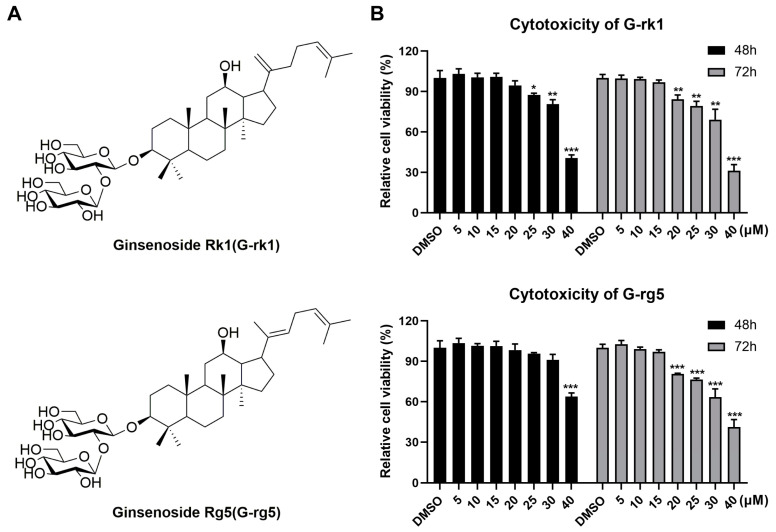
The cellular toxicities of ginsenoside rk1 (G-rk1) and rg5 (G-rg5) on A549 cells. (**A**) The chemical composition of G-rk1 and G-rg5. (**B**) G-rk1 or G-rg5 were applied to confluent A549 cells in varying concentrations. Cell viability was evaluated by the MTT assay at 48 or 72 h. Results are presented as a percentage (%) of cells that received a mock treatment (0.4% DMSO). Values are average (%) ± standard deviation (SD) of three separate assessments. * *p* < 0.05, ** *p* < 0.01, and *** *p* < 0.001 compared to the DMSO control.

To determine the safety and efficacy of G-rk1 and G-rg5 (their chemical structures are shown in Figure 1A) against PR8 infection, we first evaluated their cytotoxicity in A549 cells using the MTT assay. G-rk1 was not cytotoxic to A549 cells at doses of 20 μM after 48 h of treatment or 15 μM after 72 h of therapy (Figure 1B). A549 cells had a slightly greater safety dose for G-rg5, a structurally geometric isomer of G-rk1. No overt cytotoxicity on A549 cells was seen at concentrations of 30 μM after 48 h of treatment and 15 μM after 72 h of treatment. Based on these results, we selected 15 μM as the maximum concentration in subsequent studies. G-rk1 and G-rg5 had CC_50_ values of 34.8 and >40 μM, respectively, on A549 cells after 72 h (CC_50_ is the concentration needed to cut normal cell viability by 50%).

**Figure 2:**
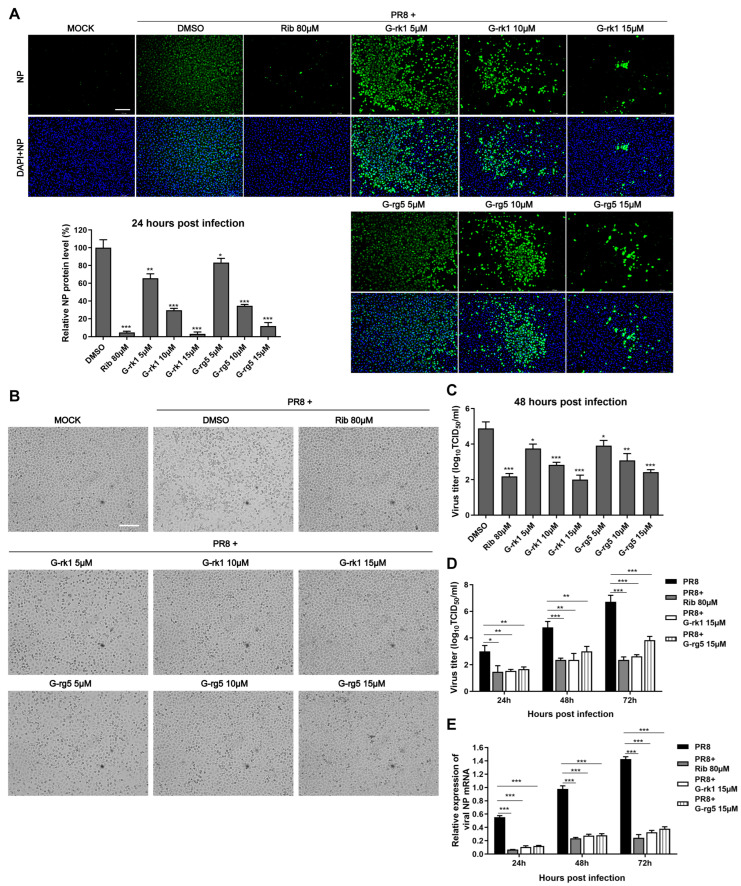
G-rk1 and G-rg5 inhibit PR8 influenza virus infection in A549 cells. Cells were cultivated in fresh media containing various concentrations of the relevant substance after being infected with PR8 (0.1 MOI) for 2 h at 37 °C. (**A**) Inhibition of G-rk1 and G-rg5 on PR8 replication in A549 cells by IFA at 24 hpi. Representative IFA images from one of the three independent experiments are displayed. The scale bar represents 250 µm. Based on the relative green fluorescence intensity of compound-treated cells to the virus control cells (DMSO), the results in the bar graphs represent normalized NP protein levels. (**B**,**C**) After being exposed to the compounds for 48 h, the cytopathic effect (CPE) was observed under a microscope (scale bar: 250 µm) (**B**) or the viral titer of each well including the cells and the supernatant was assessed using the endpoint dilution assay and expressed as log10 TCID_50_/_mL_ (**C**). (**D**,**E**) After being exposed to the compounds for the specified time intervals, the viral titer of the cells and the supernatant from each well was assessed using the endpoint dilution assay (**D**) or the relative viral NP mRNA level was determined using real-time RT-PCR. GAPDH served as a loading control, while the viral control sample treated with DMSO at 48 hpi served as a treatment (parallel) control (set as 1) (**E**). Results from samples treated with compounds and controls treated with DMSO were compared. Statistical significances are denoted by * *p* < 0.05, ** *p* < 0.01, and *** *p* < 0.001.

The antiviral activity of the two compounds was confirmed by IFA. As shown in Figure 2A, G-rk1 and G-rg5 attenuated viral NP expression dose-dependently. Noticeably, 15 μM of G-rk1 protected 95% of cells from PR8 infection. Our results showed that 80 μM of ribavirin exhibited obvious protection on PR8-infected A549 cells, as well. Cytopathic effects in G-rk1- or G-rg5-treated wells were reduced (Figure 2B), indicating the protection of the two compounds on A549 cells from PR8 infection. Additionally, we used virus titration at 48 hpi to investigate the antiviral activity of G-rk1 and G-rg5 against PR8 infection. Treatment with G-rk1 or G-rg5 greatly decreased the amount of virus (Figure 2C). Compared to the DMSO-treated control, 15 μM of G-rk1 and G-rg5 caused a 2.8 or 2.4 log reduction, respectively, in the virus titer. Next, we investigated the IAV inhibition kinetics in A549 cells with G-rk1 and G-rg5 at 15 μM by measuring virus titers at 24, 48, or 72 h post PR8 infection. At all time points, treatment of G-rk1 or G-rg5 dramatically reduced the viral titer (Figure 2D). Treatment of 80 μM ribavirin exhibited similar inhibition to that of 15 μM G-rk1 and G-rg5 at all time points. As expected, viral NP mRNA expression exhibited similar profiles to the virus titer (Figure 2E). Adding 15 µM G-rk1 or G-rg5 or 80 µM ribavirin dramatically reduced viral NP mRNA levels at all time points.

To explore whether G-rk1 and G-rg5 possess broad inhibition of various subtypes of IAV strains, their effect on H5N1- and H3N2-infected A549 cells was evaluated. Both G-rk1 and G-rg5 showed dose-dependent antiviral activity against both H5N1 and H3N2 at 48 hpi, indicating their antiviral effect was not limited to a specific subtype. By counting infected cells from IFA images, the 50% effective concentrations (EC_50_) of G-rk1 and G-rg5 against the three IAV strain infections were found to range from 6.2 to 14.8 M, and the corresponding selectivity index (SI) ranged from 2.6 to 5.6 (Table 1).

### 2.2. G-rk1 Interferes with IAV Entry

**Figure 3:**
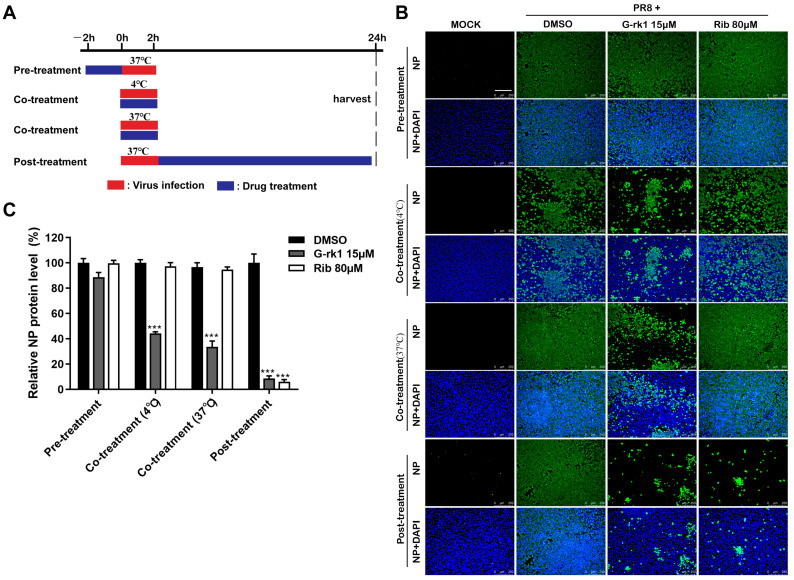
G-rk1 inhibits PR8 infection by targeting the virus entry. (**A**) Scheme for the time-course inhibition experiment. A549 cells were treated with 15 μM G-rk1 or 80 μM ribavirin for 2 h prior to PR8 (0.1 MOI) infection (pre-treatment) or incubated with G-rk1 or ribavirin during PR8 (0.1 MOI) infection at 4 °C or 37 °C for 2 h (co-treatment), or with G-rk1 or ribavirin for 24 h post-infection (0.1 MOI) (post-treatment). Paraformaldehyde was used to fix the cells, and IFA was used to show the viral NP expression at 24 hpi (**B**,**C**). Scale bar: 250 µm. Statistical significances are denoted by *** *p* < 0.001 compared to the DMSO control.

Given that G-rk1 and G-rg5 are geometric isomers, and the two compounds share very similar chemical structures and anti-IAV activities, we selected G-rk1 to investigate the antiviral mechanisms against PR8 infection in A549 cells. We conducted time-course analyses of the inhibitory effects of G-rk1 at 15 μM to determine the stage(s) of the IAV lifecycle during which G-rk1 plays its inhibitory role (Figure 3A). Pre-treating A549 cells with G-rk1 did not affect viral NP expression (Figure 3B), demonstrating that G-rk1 did not affect the cells’ sensitivity to the PR8 virus. Co-treatment of G-rk1 at 4 °C (only IAV attached to the cell surface) and 37 °C (virus attachment and internalization) led to a reduction of viral NP expression, indicating that G-rk1 may prevent virus attachment to and/or entry into cells. Viral NP synthesis was reduced by 93% when the cells were treated with G-rk1 for 24 h following PR8 infection (post-treatment), demonstrating that G-rk1 might also exert its antiviral effect when it is added during the post-virus binding stages. This is likely the result of G-rk1′s inhibiting role on the entry of the progeny virus. As expected, ribavirin’s antiviral activity was only observed post-treatment, but not in pre- and co-treatment models, as it is an RNA synthesis inhibitor. Intriguingly, the combination of 10 μM G-rk1 and 40 μM ribavirin significantly increased the inhibition of NP protein expression compared to those when either was used alone, indicating an additive or synergistic antiviral effect of G-rk1 and ribavirin against IAV replication. This finding further indicates they function at various stages of the viral lifecycle (Figure S5).

### 2.3. G-rk1 Interacts with HA via the HA1 Subunit

**Figure 4:**
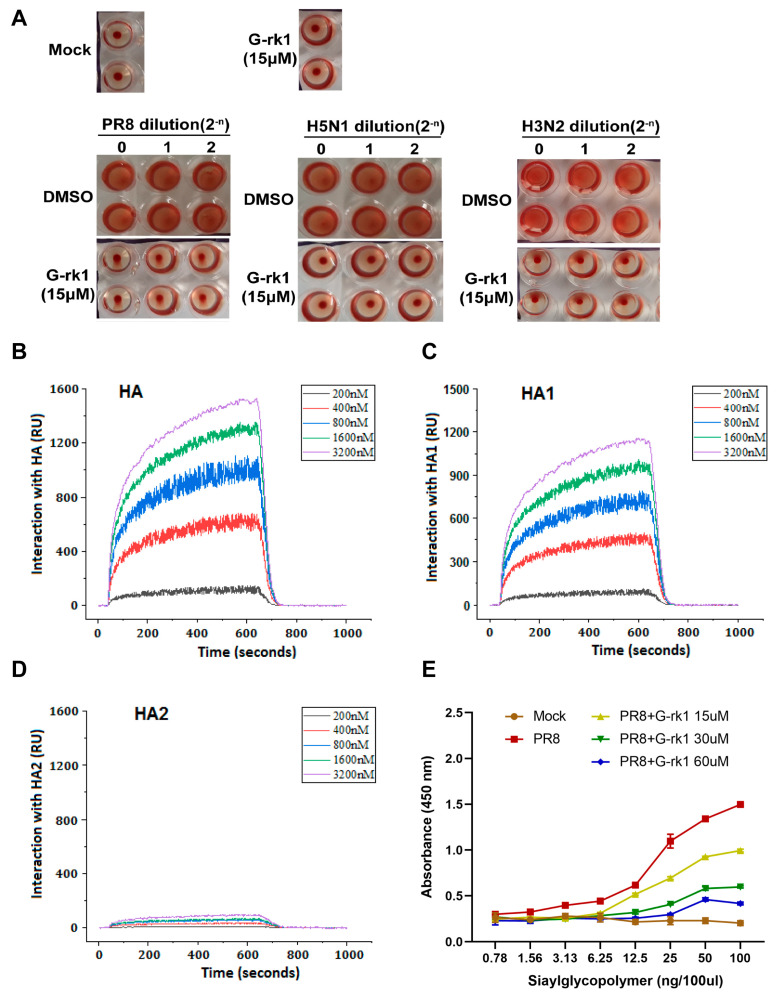
Identifying HA as the potential target of G-rk1. In (**A**), an HAI assay using 2-fold serially diluted A/Puerto Rico/8/34 (PR8, H1N1), A/Duck/Guangdong/212/2004 (H5N1), and A/Guangdong/Dongguan/1100/2006 (H3N2) viruses (staring at 16 HA units) and 1% CRBCs was performed in the absence or presence of G-rk1. In (**B**–**D**), HA or HA1 or HA2 from PR8 virus was immobilized on a photo-cross-linker sensor chip. Subsequently, G-rk1 was injected as an analyte at various concentrations, and PBST was used as the running buffer. The interaction signals were retrieved and analyzed with bScreen LB 991 software. The affinities of the interactions between G-rk1 with HA, HA1, and HA2 are shown in (**B**–**D**), respectively. In (**E**), PR8 virus (64 HA units) was treated with or without G-rkl for 2 h at 37 °C and then added into the plate coated with Neu5Acα2-3Galβ1-4GlcNAcβ-PAA-biotin (3′-SLN) at 4 °C for 12 h. The plate was then incubated with mouse monoclonal antibody against HA protein of H1 subtype at 4 °C for 3 h, and then washed with PBST and incubated with HPR-labeled goat anti-mouse IgG for 2 h at 4 °C. The absorbance values were read at 450 nm.

Binding of HA1 to the sialic acid-containing receptor mediates IAV attachment to target cells, and the membrane fusion is mainly mediated by HA2 [34]. The HAI assay has been widely used to evaluate compounds for their inhibitory effect on the interaction between HA and its cell receptor. [35]. We examined G-rk1′s suppression of IAV-mediated hemagglutination to see if HA was the target. A total of 15 μM of G-rk1 did not affect chicken red blood cells (CRBCs) settling into the bottom of assay wells without IAV, whereas 15 μM G-rk1 treatment inhibited hemagglutination caused by H1N1, H5N1, and H3N2, reflected by CRBCs settling into the bottom of assay wells (Figure 4A). These results demonstrate that G-rk1 could block the binding between HA from IAV group 1 (H1N1 and H5N1) and group 2 (H3N2) with the host cellular receptors.

The interaction between G-rk1 and HA and its subunits, HA1 or HA2 protein, was then studied using surface plasmon resonance (SPR) analysis. The results revealed that G-rk1 had a dose-dependent interaction with HA (Figure 4B) and its subunit HA1 (Figure 4C), with equilibrium dissociation constants (KD) of 14.6 nM and 93.3 nM, respectively, but it had a neglectable interaction with HA2 (KD: 0.22 M) (Figure 4D). FKBP12 (FK506 Binding Protein 1A, 12 kDa), used as an irrelevant control protein in the SPR experiment, did not interact with G-rk1. Hence, we believed that the interaction between G-rk1 and HA/HA1 was specific. These findings indicate that HA1 is the most likely target of G-rk1 for inhibiting viral attachment and cell entry.

After identifying HA (HA1) as the target, we investigated whether G-rkl had an impact on the ability of the influenza virus HA to bind to sialic acid receptors, a crucial step for viral attachment [36], via an indirect ELISA using a sialic acid-coated plate. The results showed that the PR8 virus strongly bound to the coated sialic acid receptors, which were weakened or blocked in a dose-dependent manner after the virus was treated with G-rk1 (Figure 4E). Based on these results, we conclude that G-rk1 inhibits virus attachment by blocking HA’s (H1 subtype) binding to its sialic acid receptor.

### 2.4. G-rk1 Reduces IAV-Induced Morbidity and Mortality following Lethal PR8 Infection in Mice

To evaluate G-rk1′s anti-IAV effects in vivo, we first determined the safe dose of G-rk1 in mice by intragastric administration and intranasal inoculation. The results showed that all doses administered via either route were tolerated, and the mice did not exhibit obvious weight loss or any other adverse symptoms (piloerection, altered respiratory rates, alopecia, signs of hunching, or unresponsiveness) (Figure S6). The 100 mg/kg/d of G-rk1 via intragastric administration (i.g.) or 25 mg/kg/d of G-rk1 via intranasal inoculation (i.n.) in BALB/C mice for 6 consecutive days were both tolerated well. Therefore, these doses were chosen as the maximal drug dose for in vivo anti-IAV evaluations. BALB/C mice intranasally infected with a dose of 5 × LD_50_ of PR8 were administrated by intragastric or intranasal inoculation with G-rk1 once daily for 6 consecutive days, starting on day 1 before infection.

**Figure 5:**
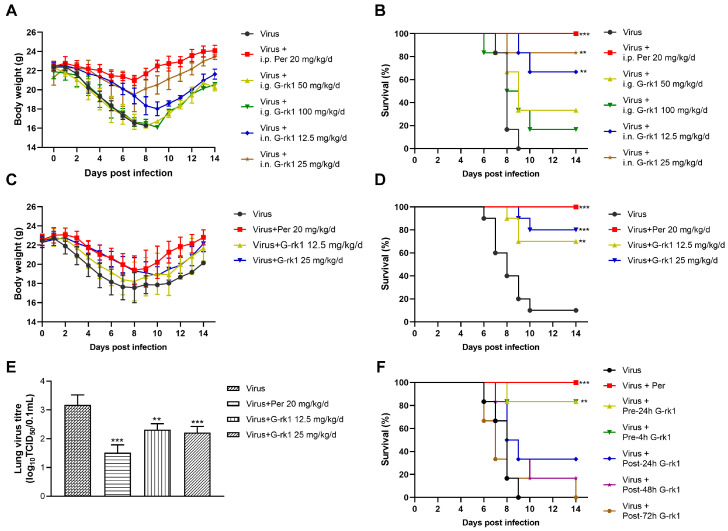
G-rk1 treatment decreases BALB/C mice’s morbidity and mortality from PR8 infection. In (**A**–**E**), BALB/C mice were infected with 5 × LD_50_ of PR8 and treated with G-rk1 via intragastric administration (i.g.) or intranasal inoculation (i.n.) or with PBS as a control (once daily for a consecutive 6 days starting a day before infection). Peramivir, a positive control, was administered daily via intraperitoneal injection (i.p.). Daily assessments were conducted for weight loss (**A**,**C**) and mortality (**B**,**D**) (n = 6 for (**A**) and (**B**); n = 10 for (**C**,**D**), i.n). In (**E**), the effect of G-rk1 administered intranasally on lung virus titers in PR8-infected mice at 4 dpi (n = 5) is shown. In (**F**), BALB/C mice were infected with 5 × LD_50_ of PR8, and the initial 25 mg/kg/d of G-rk1 was intranasally introduced either at 24 (Pre-24 h) or 4 h (Pre-4 h) prior to or 24 (Post-24 h), 48 (Post-48 h), or 72 h (Post-72 h) post-infection, respectively, followed by G-rk1 treatment for 6 consecutive days. Survival rates in all groups were monitored daily for 14 days. Statistical significances are denoted by ** *p* < 0.01, and *** *p* < 0.001 compared to Virus Control.

Figure 5A,B show that intranasally administrated G-rk1 protected mice from PR8 infection, significantly reflected by decreased weight loss and increased survivals, 66.7% (4/6) and 83.3% (5/6) in G-rk1-treated mice at 12.5 and 25 mg/kg/d, respectively, as no mouse survived in the PR8-infected untreated group. However, G-rk1 administered via the intragastric route showed much lower protection, only 33.3% (2/6) and 16.7% (1/6) survivals in mice treated with 50 and 100 mg/kg/d, respectively. Therefore, intranasal inoculation was identified as the more effective route for G-rk1 administration and was applied in further G-rk1 evaluations against PR8 infection. Figure 5C confirms that intranasal administration of 25 mg/kg/d and 12.5 mg/kg/d of G-rk1 resulted in significantly reduced mortality compared to the PBS control, with 80% (8/10) and 70% (7/10) survival, respectively, while only 10% of PR8-infected mice survived without treatment. This was accompanied by a comparable decrease in weight loss following PR8 infection in mice treated with 25 and 12.5 mg/kg/d G-rk1 (Figure 5D). On day 4 after PR8 infection, G-rk1 treatment reduced total lung tissue virus titers, consistent with the in vitro results (Figure 5E). Peramivir, one of the IAV neuraminidase inhibitors widely used clinically, was used as the positive control in our in vivo studies, and 20 mg/kg/d of peramivir showed 100% protection of PR8-infected mice. Significant reductions in weight loss and lung tissue viral titers following PR8 infection were also observed in the peramivir-treated mice. Our data suggest that G-rk1 by intranasal inoculation robustly protects mice from lethal IAV infection.

To investigate whether delayed G-rk1 administration would affect its protection for IAV-infected mice, which might mimic early clinical treatment, 25 mg/kg/d of G-rk1 was given to mice by intranasal inoculation at 24 or 4 h prior to or 24, 48, or 72 h post PR8 virus infection, followed by G-rk1 treatment for 6 consecutive days. The results showed that delayed G-rk1 administration sharply decreased the protection of PR8-infected mice. Overall, 83.3% (5/6) of mice treated with G-rk1 initiated at 24 h or 4 h prior to virus infection survived, while among those initiated at 24, 48, and 72 h post-infection, only 33.3% (2/6), 16.7% (1/6), and 0% (0/6) survived, respectively (Figure 5F). These results suggest that early intervention with G-rk1 is crucial for a maximal protecting effect on IAV-infected mice.

## 3. Discussion

White ginseng (WG, dried ginseng), red ginseng (RG, dried and steamed ginseng), and black ginseng (BG, dried and steamed several times) are the three most used species for commercial ginseng products [37]. Many studies have reported that BG possesses more potent biological activities than RG and WG [38,39], and the absorption rate of ginsenosides in BG is higher in healthy adults [40]. Eun-Ha Kim et al. reported that RG extract was inferior to BG extract in inhibiting A(H1N1) pdm09 infection, likely due to BG’s higher contents of functional ginsenosides, such as rg3, rk1, and rg5 [41]. However, the substance(s) that exert the crucial antiviral activity in BG are still unknown. In this study, we assessed the anti-IAV effects of 23 ginsenosides and discovered that G-rk1 and G-rg5 have considerable effects against different IAV infections. We have shown that G-rk1 inhibits viral entry by interfering with the binding of HA to its sialic acid receptor. In vivo, intranasal injection of G-rk1 therapy decreased IAV PR8-induced weight loss and mortality in infected mice.

Since the initial step in the viral replication cycle is viral attachment onto the target cells, preventing viral attachment and entry would prevent viral infection. Seven entry inhibitors have been authorized to treat clinical infectious illnesses caused by RSV, HIV, and herpes simplex virus (HSV) [42]. The sialic acid receptor for HA1 mediates IAV attachment to target cells. Next, the low pH (5.5 to 5.0) of the endosome triggers HA conformational change, leading to the fusion of the virus envelope and the endosomal membrane [43]. Among licensed anti-IAV drugs, arbidol is the only viral entry inhibitor inhibiting IAV replication by blocking viral membrane fusion. However, it is only approved for clinical use in Russia and China [44]. Some compounds were documented to block IAV binding to host cells, including teicoplanin derivatives [45], neoechinulin B and its analogs [46], and aureonitol [47]. However, these results were obtained heavily depending on HAI assay and needed more direct demonstration and in vivo study. In our study, G-rk1 and G-rg5 suppressed three different IAV infections in A549 cells with a low EC_50_ ≤ 14.8 µM, a much lower concentration compared with that from the reported G-rb1 (450 µM) [31], whereas 10 µM of G-rb1 did not exhibit any inhibition of PR8 replication in A549 cells in our antiviral compound screening. G-rk1 at 15 µM robustly inhibited three IAVs’ adsorption to CRBCs. Furthermore, our results revealed that G-rk1 binds tightly to the HA and HA1, which blocks IAV binding to the cellular sialic acid receptor in a dose-dependent manner, whereas it has little interaction with HA2 (Figure 4). These results demonstrate that G-rk1 acts as a potent IAV entry inhibitor by targeting HA1 rather than HA2.

It has been demonstrated that combining inhibitors with different mechanisms increases their antiviral effect and reduces drug resistance [17]. As expected, the combination of G-rk1 with ribavirin, a well-known viral RNA synthesis inhibitor, resulted in a stronger antiviral effect than either compound used alone (Figure S5), suggesting that G-rk1 could be a novel compound used in combination with other anti-IAV drugs.

From our in vivo studies, intranasally delivered G-rk1 showed marked antiviral activity against PR8 infection, leading to decreased weight loss and mortality, with an 80% survival rate. However, G-rk1 administrated intragastrically showed minimum protection. The discrepancy between the two routes could be attributed to poor oral bioavailability. It was reported that the bioavailability of 50 mg/kg G-rk1 after oral administration was only 4.23%, which might be caused by poorer intestinal mucosal permeability and potentially altered compound stability in the gastrointestinal tract [48,49].

Importantly, although some mice treated with G-rk1 at 24 or 48 hpi were protected, mice treated with G-rk1 even just 4 h before infection showed significantly higher survival rates, indicating that G-rk1 is most suitable for prevention. The good protection of G-rk1 administrated by intranasal inoculation on PR8-infected mice suggests that it could potentially be developed into an anti-influenza aerosol, a common and practical drug administration strategy for respiratory infectious diseases in the clinic [50]. For instance, inhaled ribavirin decreases the progression to lower respiratory tract infections and mortality in immunocompromised patients [51,52]. Therefore, it is possible to use G-rk1 in an inhalation, alone, or combined with other anti-IAV drugs to treat influenza patients during the early infection.

## 4. Materials and Methods

### 4.1. Cell Lines and Virus Strains

We purchased Madin-Darby canine kidney cells (MDCK cells) and A549 human lung cancer cells (A549 cells) from the Center of Cellular Resource, Chinese Academy of Sciences (Shanghai, China). The cells were cultured in a 37 °C, 5% CO_2_ incubator in Dulbecco’s Modified Eagle’s Medium (DMEM, Gibco, NY, USA), supplemented with 10% fetal bovine serum (FBS), 100 U/mL of penicillin, and 100 g/mL streptomycin.

The Chinese Center for Disease Control and Prevention provided the H1N1 IAV strain A/Puerto Rico/8/34 (H1N1, PR8) and H3N2 IAV strain A/Guangdong/Dongguan/1100/2006 viruses (Beijing, China). The Veterinary Technology Center of South China Agricultural University generously donated avian IAV strains A/Duck/Guangdong/212/2004 (H5N1) (Guangzhou, China). For 48 h, virus stocks were passaged in 10-day-old chicken embryonated eggs. After harvesting the allantoic fluid, aliquots were kept at −80 °C until needed. Utilizing the endpoint dilution test as previously published, viral titers were calculated as the 50% tissue culture infectious dose (TCID_50_/_mL_) in confluent MDCK cells in 96-well microtiter plates [53]. A biosafety level 3 lab was used for all experiments involving H5N1 virus strains.

### 4.2. Compounds

Ginsenosides rk1 (G-rk1) and rg5 (G-rg5), and 21 other ginsenosides with HPLC purities ≥98%, were bought from Chengdu Gelipu Biotechnology Co., Ltd. in Chengdu, China. With a purity of 99%, ribavirin hydrochloride (Rib) was bought from Guangdong Star Lake Bioscience Co., Ltd. in Zhaoqing, China. Guangzhou Nucien Pharmaceutical Co., Ltd. sold peramivir in a sterile 0.9% NaCl solution (0.3 g/100 mL) (Guangzhou, China). G-rk1, G-rg5, and 21 other ginsenosides were dissolved in dimethyl sulfoxide (DMSO) at a concentration of 30 mM as stock solutions for in vitro tests. Ribavirin hydrochloride was dissolved in PBS. For intranasal inoculation, G-rk1 was first dissolved in DMSO to a 250 mg/mL stock solution and then diluted with PBS to a 25 mg/mL work solution for nasal drop use. For intragastric administration, G-rk1 was prepared as a 10 mg/mL concentration in a 0.3% sodium carboxymethyl cellulose solution. Peramivir solution was intraperitoneally injected after a proper dilution with PBS.

### 4.3. Mice

We purchased female BALB/C mice from Jinan Pengyue Medical Laboratory Animal Ltd. (Jinan, China). Mice were kept in special pathogen-free isolators (SPF). According to the regulations for the care and use of animals for scientific reasons, experiments were carried out on mice that were 6 to 8 weeks old and were approved by South China Agricultural University’s Institutional Animal Care and Use Committee.

### 4.4. Cytotoxicity Assay

An MTT test was used to measure the viability of A549 cells in the presence of various doses of G-rk1 or G-rg5. To achieve 100% confluency, the cells were cultured in 96-well plates for 24 h at 37 °C. Fresh medium was added, and serially diluted compounds were incubated at 37 °C for 48 or 72 h. After that, the cells were stained with a 0.5 mg/mL solution of 3-(4,5-dimethylthiozol-2-yl)-3,5-diphenyl tetrazolium bromide (MTT; Sigma-Aldrich, Saint. Louis, MO, USA). The cell viability index was calculated using the mean optical density (OD) readings from six replicated wells for each treatment. Using GraphPad Prism 8.0, the 50% cytotoxic concentration (CC_50_) was determined (GraphPad Software, San Diego, CA, USA).

### 4.5. Indirect Immunofluorescence Assay (IFA)

We utilized an indirect immunofluorescence technique to quickly assess drugs’ antiviral efficacy against IAV infection. IAV-infected or uninfected cells were briefly fixed with 4% paraformaldehyde for 15 min, followed by permeabilization with 0.3% Triton X-100 for 10 min at room temperature (RT) and blocked with 5% bovine serum albumin (BSA) for 60 min at 37 °C. Anti-NP antibodies (1:500 dilution, Sino Biological, Beijing, China) were then incubated with the cells overnight at 4 °C, after 3× washes with PBS, and the anti-mouse IgG antibody coupled with Alexa Fluor^®^ 488 (green) (Cell Signaling Technology, Danvers, MA, USA) was added to the cells for 1 h at 37 °C, followed by 3x PBS washes. Finally, 50 μL of 4, 6-diamidino-2-phenylindole was used to stain the cells’ nuclei (DAPI, 300 nM; Sigma-Aldrich, Saint. Louis, MO, USA). A Leica DMI 4000B fluorescence microscope was used to record immunofluorescence (Leica, Wetzlar, Germany). The infection rate was defined as the proportion of infected cells to all cells. The ratio of the infection rate in the compound-treated groups to that in the DMSO-treated control was used to calculate the relative infected-cell percentage. Using the GraphPad Prism 8.0 software, the relative infected-cell percentage was plotted as a function of compound concentration to get the EC_50_ value (the concentration necessary to protect 50% of cells from IAV infection).

### 4.6. Viral Inhibition Assay

A549 monolayers were first infected by IAV. Cells were then treated with DMEM containing ranged doses of the test compound after removing supernatants containing unbound viral inoculums. At indicated time points, the cells were collected and underwent three freeze–thaw cycles at −80 °C and 4 °C to release cellular virions. The final virus titers of cells and supernatants were calculated and expressed as log10 TCID_50_/_mL_ using MDCK cells in an endpoint dilution test.

### 4.7. Real-Time Reverse-Transcription PCR (RT-PCR)

Following the manufacturer’s instructions, total RNA from the A549 cells was extracted using a total RNA quick extraction kit (Fastagen, Shanghai, China) and reverse-transcribed into cDNA using a reverse transcription kit (TaKaRa, Kyoto, Japan). Using the CFX96 Real-time PCR machine, 2 RealStar Green Power Mixture (including SYBR Green I Dye) (Genstar, Beijing, China) was used to perform real-time quantitative reverse transcription PCR (qRT-PCR) (Bio-Rad, California, USA). The following are the sense and anti-sense primer sequences: NP (5′-ACCAGAAGATKTGTCMTTCCAGGG-3′ and 5′-TACTCCTCCGCATTGTCTCCGAAG-3′); GAPDH (5′-GCACCGTCAAGGCTGAGAAC-3′ and 5′-TGGTGAAGACGCCAGTGGA-3′). Relative mRNA expression was calculated by the 2^−ΔΔCT^ method [54], and *GAPDH* expression served as the endogenous control.

### 4.8. Time-Course Inhibition Assay

A549 cells were infected by IAV PR8 in 24-well plates for 2 h at 37 °C. In pre-treatment, cells were incubated with the appropriate compound for 2 h at 37 °C, washed three times with PBS, and then exposed to PR8 for 2 h at 37 °C. In the co-treatment, cells were treated with compound and infected with PR8 simultaneously for 2 h at 4 °C or 37 °C, respectively, and then washed three times with PBS. In post-treatment, cells were first infected with PR8 for 2 h at 37 °C, followed by three washes with PBS, and then incubating in fresh medium containing the compound. At 24 hpi, the virus infection level in the treated cells was assessed using IFA, as mentioned above.

### 4.9. Hemagglutination Inhibition Assay (HAI)

We used a hemagglutination assay to determine whether G-rk1 inhibited HA-mediated hemagglutination of chicken red blood cells (CRBCs). In total, 25 μL G-rk1 (5 μM or 15 μM) were mixed with 25 μL serially diluted influenza virus at RT for 30 min, then 50 μL 1%CRBCs was added and incubated for 15 min at 37 °C. The results were recorded by taking images of the hemagglutination in the plates.

### 4.10. Surface Plasmon Resonance (SPR) Analysis

A Berthold bScreen LB 991 (LabXMedia company, Midland, Canada) was used to examine the interactions between IAV HA or HA1, or HA2 and G-rk1 at 4 °C. IAV strain A/Puerto Rico/8/1934 (H1N1) HA protein or its subunit HA1 or HA2 (Sino Biological Inc, Beijing, China) were immobilized on a sensor chip (Photo-cross-linker SensorCHIPTM) using an amine coupling kit (GE Healthcare, Buckinghamshire, UK). Then, using PBST (10 mM phosphate buffered salt solution containing 0.1% Tween 20, pH 5.0) as the running buffer, the chemical was administered as analytes at varied concentrations, with contact times of 600 s and dissociation times of 360 s. After each test cycle, the chip platforms were cleaned with regeneration buffer (Glycine-HCl, pH 2.0). Using the data analysis software of the bScreen LB 991 unlabeled microarray system, the process and analysis of association and dissociation rate constants (Ka/Kon and Kd/Koff, respectively), as well as the equilibrium dissociation constant (kD, kd/ka), were calculated following a single-site binding model (1:1 Langmuir binding), with mass transfer limitations for binding kinetics determination.

### 4.11. The Influence of G-rk1 on Sialic Acid Receptor Binding Ability of IAV by an Indirect ELISA

Using an indirect ELISA, as previously mentioned, the receptor binding capacity of PR8 treated with varied doses of G-rkl was determined [55]. In brief, 100 μL of 5 μg/mL streptavidin was added to each well of a 96-well plate, and the plate was rinsed three times with PBS before being incubated at 4 °C for 16 h. The 96-well plate was coated with the sialic acid receptors, Neu5Ac2-3Gal1-4GlcNAc-PAA-biotin (3′-SLN) (GlycoTech Inc., Gaithersburg, MD, USA), which had been diluted with PBS. The plate was then incubated at 4 °C for 12 h, after which the supernatant was discarded. The plate was then blocked with 5% skim milk for 8 h at 4 °C, after which the supernatant was removed, and was washed three times with PBST. G-rkl was applied to the PR8 of 64 hemagglutinin units at doses of 0, 15, 30, and 60 μM for 2 h each at 37 °C. Both treated and untreated viruses were added into the sialic acid-coated plate. The plate was then incubated at 4 °C for 12 h, washed, and anti-HA mouse monoclonal antibody (clone M100016) (Zoono-gene, Beijing, China, 1:1800) was added for 60 min. The plate was again washed three times with PBST, and HPR-labeled goat anti-mouse IgG (1:12,000) was added for 2 h at 4 °C before being washed with PBS, and tetramethylbenzidine-H_2_O_2_ substrate solution (Solarbio, Beijing, China) was then added and left at room temperature for 10 min. Then, 0.2 M H_2_SO_4_ was added to the plate, and the OD values at 450 nm were measured on a plate reader.

### 4.12. In Vivo Viral Challenge and G-rk1 Treatment

In lethal PR8 infections, BALB/C mice were intranasally infected with 5 times LD_50_ of the PR8 virus (in 30 μL PBS) under methoxyflurane anesthetization. G-rk1 was administered gavagely or intranasally. G-rk1 administration began 24 h before the infection and continued for 6 consecutive days. A positive control compound, peramivir, was injected intraperitoneally at the indicated doses. Each mouse underwent daily weight and mortality assessments.

### 4.13. Lung Tissue Viral Titers

After being taken out of the mouse, the lungs were weighed, cleaned in PBS, homogenized in 1 mL of PBS with 100 U/mL penicillin and 100 g/mL streptomycin, and then centrifuged at 3600× *g* for 5 min at 4 °C. After that, supernatants were collected for assessing the virus titer in MDCK cells using the endpoint dilution test, as previously mentioned.

### 4.14. Statistical Analysis

All values are shown as the mean SD of at least three experiments. When just two groups were compared, the Student’s *t*-test was used to evaluate the statistical significance; when more than two groups were compared, a one-way analysis of variance (ANOVA) was used. To conduct the statistical analysis, GraphPad Prism 8 was used (GraphPad Software, San Diego, CA, USA). * *p* < 0.05, ** *p* < 0.01, and *** *p* < 0.001 were considered statistically significant at different levels. The survival curves were plotted in GraphPad Prism 8 and compared with a log-rank (Mantel–Cox) test.

## 5. Conclusions

In summary, our study revealed that, among the evaluated 23 ginsenosides, G-rk1 and G-rg5 showed the strongest inhibition of infected A549 cells by various IAV strains. Mechanistically, G-rk1 interferes with IAV’s attachment to its host cells via binding to HA1, thus blocking the binding of HA to its sialic acid receptors. In our in vivo study, intranasal inoculation of G-rk1 protected mice from a lethal IAV PR8 challenge, and early intervention with G-rk1 was found to have better antiviral outcomes. Our findings show for the first time that ginseng-derived G-rk1 binds to HA1. This study provides new insights into the potential of G-rk1 as a novel entrance inhibitor, which might be used alone or in combination with other inhibitors to prevent and/or treat IAV infections.

## Figures and Tables

**Table 1:** Cellular toxicity and inhibitory effects of G-rk1 and G-rg5 against IAV in A549 cells.

Compounds	^a^ CC_50_ (μM)	H1N1 (PR8)		H5N1		H3N2	
		^b^ EC_50_ (μM)	^c^ SI	^b^ EC_50_ (μM)	^c^ SI	EC_50_ (μM)	^c^ SI
G-rk1	34.8	6.2	5.6	10.4	3.3	13.5	2.6
G-rg5	>40.0	8.2	>5.0	14.8	>2.8	11.5	>3.6

^a^ CC_50_ is a concentration needed to cut normal cell viability by 50%; ^b^ EC_50_ is a concentration needed to protect 50% of cells from IAV infection; ^c^ SI (selectivity index) is the ratio of CC_50_ to EC_50_.

## Data Availability

All data related to this study are included in the article or uploaded as supplementary information. Other data can be obtained from the corresponding author if reasonably requested.

## Supplementary Materials

The supporting information can be downloaded at: https://www.mdpi.com/article/10.3390/ijms24054967/s1.
