# Supplementary material for: Antiviral Effect of Ginsenosides rk1 against Influenza a Virus Infection by Targeting the Hemagglutinin 1-Mediated Virus Attachment
Source: Int J Mol Sci. 2023 Mar 4;24(5):4967. doi: 10.3390/ijms24054967 (PMC10003360; doi:10.3390/ijms24054967)
Supplement: Supplementary file 1 [file ijms-24-04967-s001.zip › ijms-2210911-supplementary.pdf]

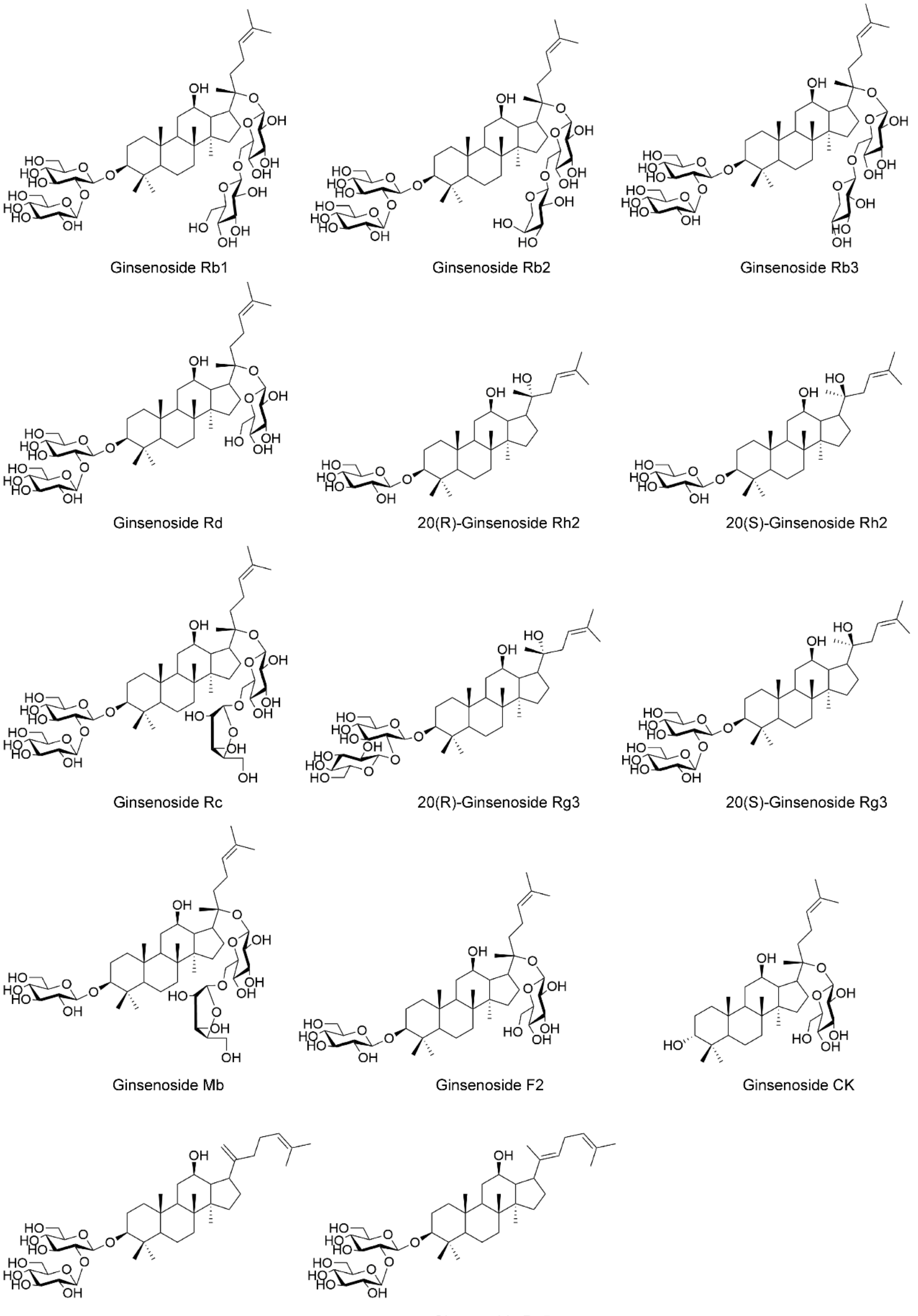

Figure S1. Chemical structures of protopanaxadiol saponins (PPD).

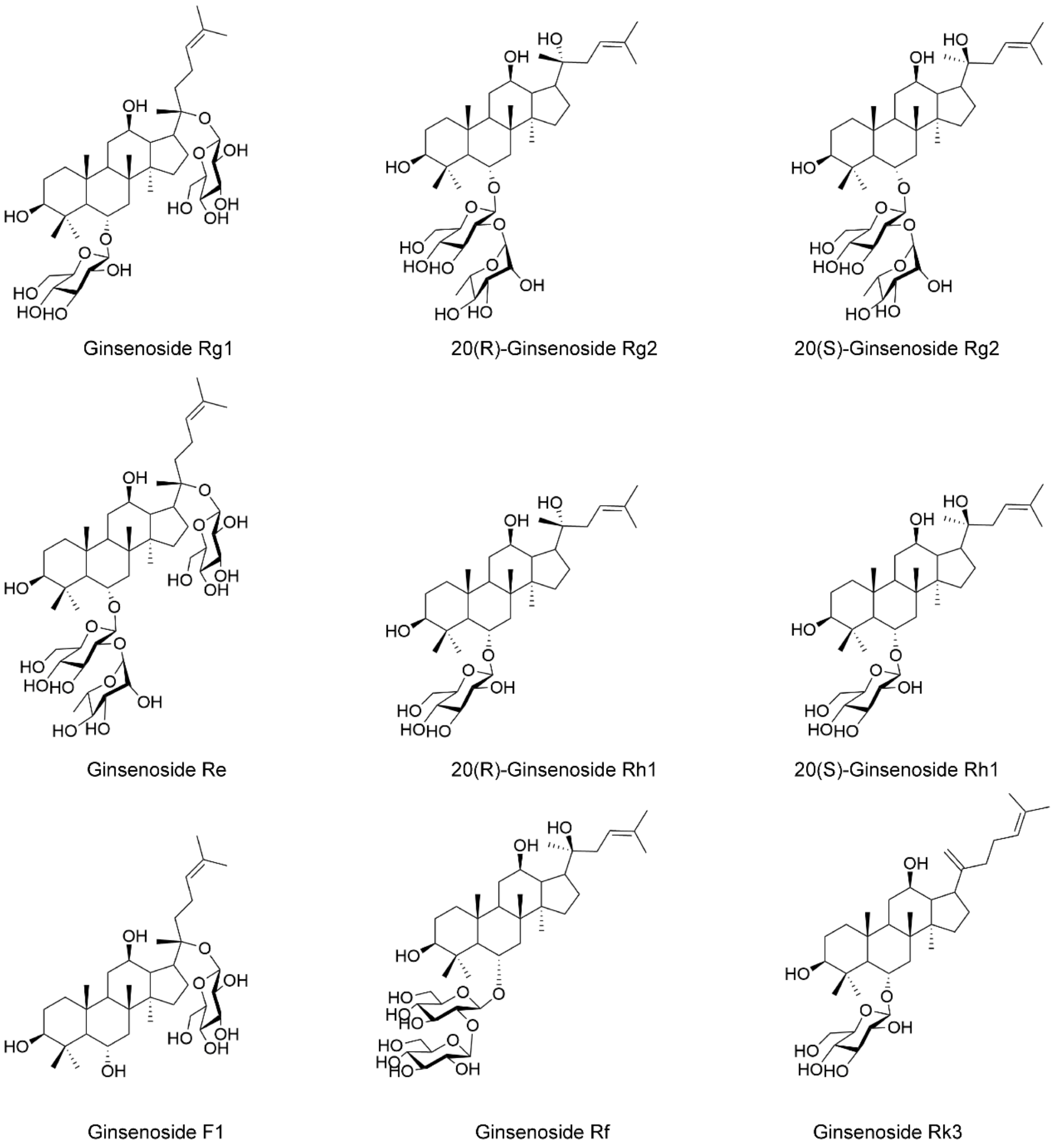

Figure S2. Chemical structures of protopanaxatriol saponins (PPT).

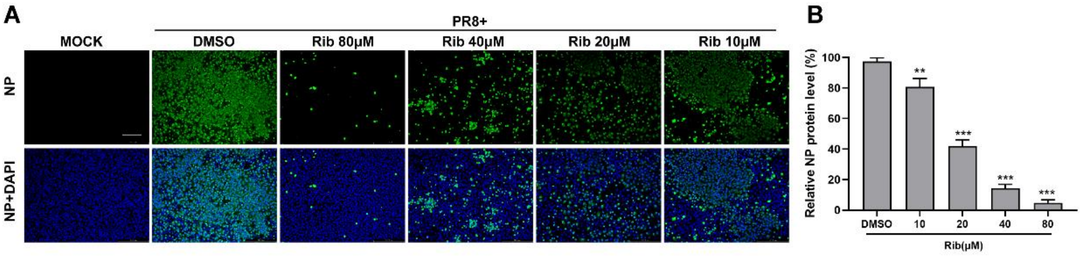

Figure S3. The antiviral effect of ribavirin against PR8 virus replication. A549 cells were infected with PR8 virus (0.1 MOI) for 2 h at 37°C and then cultured in fresh medium containing ribavirin. At 24 hpi, the cells were subjected to IFA. Representative IFA images are shown in **A**. Scale bar: 250 μm. Results shown in **B** are normalized NP protein levels based on the percentage of infected cell counts among total cell counts. Statistical significances are denoted by $^{**}p < 0.01$ and $^{***}p < 0.001$ compared to DMSO groups.

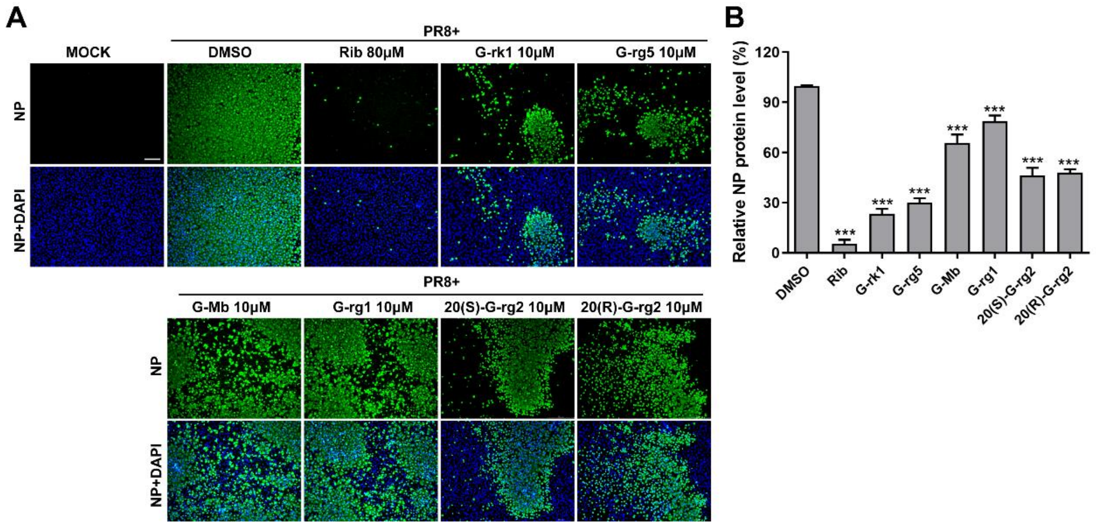

Figure S4. Antiviral activities of different Ginsenosides against PR8 (H1N1) infection in A549 cells. Cells grown in 96-well plates were infected with PR8 strain (0.1 MOI) for 2 h at 37°C and then cultured in fresh medium containing 10 μM of ginsenosides or 80 μM of ribavirin (Rib). At 24 hpi, the cells were fixed with paraformaldehyde and the viral NP expression was detected by indirect immunofluorescence assay (IFA) (**A**). Scale bar: 250 μm. Results are normalized NP protein levels based on the percentage of infected cell counts among total cell counts (**B**). Statistical significances are denoted by ***$p < 0.001$ compared to DMSO control.

Table S1. Cellular toxicity and inhibitory effects of G-Mb, G-rg1, 20(S)-G-rg2, 20(R)-G-rg2 and Rib against IAV PR8 in A549 cells.

**Compounds**	**[a]CC_{50} (μM)**	**[b]EC_{50} (μM)**	**[c]SI**
G-Mb	41.0	>20	<2.05
G-rg1	36.9	>10	<3.7
20(S)-G-rg2	37.87	15.6	2.43
20(R)-G-rg2	33.6	>10	<3.4
Rib	>300	17.3	>17.3

[a] CC_{50} is a concentration needed to cut normal cell viability by 50%; [b] EC_{50} is a concentration needed to protect 50% of cells from IAV infection; [c] SI (selectivity index) is the ratio of CC_{50} to EC_{50}.

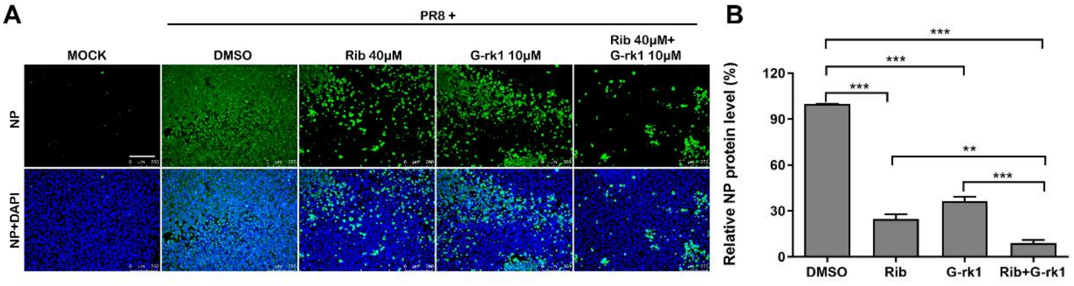

Figure S5. The synergistic effect of G-rk1 and ribavirin against PR8 virus replication. A549 cells were infected with PR8 virus (0.1 MOI) for 2 h at 37°C and then cultured in fresh medium containing G-rk1 or ribavirin or their combination. At 24 hpi, the cells were analyzed by IFA. Representative IFA images from one of the three independent experiments are shown in **A**. Scale bar: 250 μm. Results shown in **B** are normalized NP protein levels based on the percentage of infected cell counts among total cell counts. Statistical significances are denoted by **$p < 0.01$ and ***$p < 0.001$ between indicated groups.

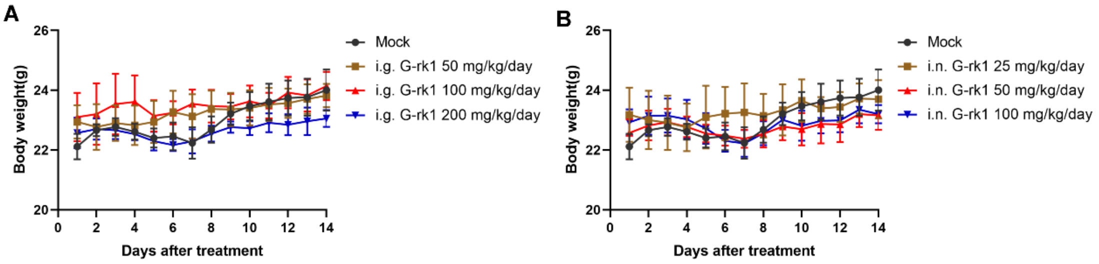

Figure S6. Toxicity of G-rk1 in mice by intragastric administration (i.g.) (**A**) and intranasal inoculation (i.n.) **(B)**, respectively. BALB/C mice (n = 8) were treated with G-rk1 once daily for 6 consecutive days. Body weights in each group were monitored daily for up to 14 days after the first treatment.
